# Individual and social determinants of COVID-19 vaccine hesitancy and uptake in Northwest Syria

**DOI:** 10.1186/s12913-024-10756-z

**Published:** 2024-03-01

**Authors:** Orwa Al-Abdulla, Maher Alaref, Agneta Kallström, Jussi Kauhanen

**Affiliations:** 1 Institute of Public Health and Clinical Nutrition, Faculty of Health Sciences, The University of Eastern Finland, 70211 Kuopio, P.O. Box 1627, Finland; 2 Strategic Research Center (Öz SRC), Incili Pinar MAH, Gazi Muhtar Paşa BUL, Doktorlar Sitesi, 38E, 104, 27090 Sehitkamil, Gaziantep, Türkiye

**Keywords:** COVID-19, Health, Hesitancy, Syria, Vaccine

## Abstract

**Introduction:**

The COVID-19 outbreak devastated the fragmented health system in Syria, a war-torn country, and exaggerated the demands for humanitarian assistance. COVID-19 vaccination was rolled out in Northwest Syria, an area out of government control, in May 2021. However, vaccine acceptance rates are still minimal, which is reflected in the meager percentage of vaccinated people. The study aims to investigate the effectiveness of the humanitarian actors’ plans to address the COVID-19 vaccine hesitancy and conclude practical strategies for boosting vaccine uptake in Northwest Syria.

**Methods and materials:**

Two questionnaires were developed to collect data from humanitarian organizations involved in the COVID-19 vaccination campaign and people from northwest Syria. Data analysis was performed using SPSS 22 data analysis program.

**Results:**

According to the findings, 55.5% of people refused the COVID-19 vaccine. The results showed a knowledge gap and lack of evidence regarding humanitarian actors’ strategies to address the vaccine’s low uptake. Besides, it was found that doctors and medical workers were reliable sources of information about the vaccine. However, they were not systematically engaged in community mobilization and risk communication to promote people’s perspectives on the vaccine.

**Conclusion:**

Risk communication and community engagement programs were not significantly associated with increasing the COVID-19 acceptance rate. Humanitarian actors must reconsider their strategies to address vaccine hesitancy in Northwest Syria. These strategies should engage medical professionals through dialogue sessions on the realities of the pandemic and vaccine development mechanism based on a compelling and evidence-based approach.

**Supplementary Information:**

The online version contains supplementary material available at 10.1186/s12913-024-10756-z.

## Introduction

The war in Syria has divided the country into three political areas with different health governance structures. The Northwest Syria NWS region, comprising parts of Aleppo and Idleb governorates and home to over 4.5 million people of which more than half are Internally Displaced Persons IDPs, is governed in the north by the Syrian National Army, backed by the Turkish government. The western part of the region is controlled by the Syrian Salvation Government affiliated with Hay’at Tahrir al-Sham (HTS) [1–3]. The protracted war since 2011 and damaged infrastructure in Syria left millions with urgent needs for essential services, including medical care, water and sanitation, food, and shelter [4]. The humanitarian organizations and United Nations UN agencies initiated cross-border operations from neighborhood countries, like Türkiye, to respond to the people’s needs in NWS, pursuant to the United Nations Security Council Resolution UNSCR 2165 (2014) [5–7]. However, violence against health care, politicization, displacement, low-income rates, and socioeconomic factors have hampered efforts to respond adequately to humanitarian needs [8, 9].

The rapid spread and high mortality rate of the COVID-19 outbreak caused severe disruptions to the health systems worldwide, especially in war-affected countries such as Syria [10, 11]. The COVID-19 outbreak devastated the fragmented health system in Syria and exaggerated the demands for humanitarian assistance [12]. As of July 2023, a total of 106,451 cases have been documented in NWS, and the cumulative count of fatalities stands at 2,527, with a gender distribution of 59% male and 41% female [13]. Along with the high number of cases, a pattern of sharp inequalities and poor community engagement regarding COVID-19 response has been reported [14, 15]. World Health Organization WHO and the Health Cluster in Gaziantep– Türkiye (NWS response) have developed a decentralized nine-pillar response plan to the COVID-19 outbreak in NWS. Nonetheless, several obstacles present themselves in the form of case management, Risk Communication and Community Engagement RCCE, and adherence to COVID-19 preventive measures [16, 17]. COVID-19 vaccination was rolled out in NWS in May 2021 by WHO and UNICEF in collaboration with Syria Immunization Group SIG [18, 19]. However, vaccine acceptance rates in Syria are still minimal, which is reflected in the meager percentage of vaccinated people [20]. Based on information released by the Health Cluster for NWS response, as of July 2023, less than 18% of the total population had received full COVID-19 vaccination [21]. In a previous study, we found that there is a dearth of studies on COVID-19 vaccination in NWS [22], which might be a reason that is impeding practical progress in enhancing the vaccination rates among people in NWS.

### Vaccine hesitancy and response strategies

Vaccine hesitancy is defined as a refusal or reluctance to accept vaccination despite the availability of vaccination services [23]. COVID-19 vaccine acceptance rates in low and middle-income countries are noticeably poor [24]. Information deficits, low levels of education and public health awareness, ineffective governmental efforts, and disinformation and rumors contributed to public mistrust and perceived threats regarding the COVID-19 vaccine [25]. Acharya et al. argued that political instability, information deficits, vaccine mistrust, and poor income rates affect access to vaccine services and consequently increase hesitancy in low-income countries, such as Syria [26]. Similarly, a study about the COVID-19 vaccine in Syria found that the COVID-19 vaccine hesitancy rate is very high compared with other countries due to the lack of knowledge about the presence of vaccination campaigns and low health awareness [27]. As of April 2023, 16.5% of the total population in NWS received at least one dose of the vaccine (partially vaccinated), and only 9.9% are fully vaccinated (at least two doses) [28]. In a recent study, Karaca and Çelik found that 42% of people in NWS refused the COVID-19 vaccine, and 15.1% said they were hesitant about accepting the COVID-19 vaccine. The main reasons for rejecting the vaccine were related to the low perceived benefits from the vaccine, health concerns related to adverse effects, and low subjective risk. Social norms and religious beliefs were not associated with vaccine rejection [29]. According to Mohamed et al., health concerns due to side effects and conspiracy beliefs– such as the notion that COVID-19 was created by vaccine manufacturers to promote vaccine sales, particularly given the proximity of vaccine rollout to the disease’s emergence– are related to the low COVID-19 vaccine acceptance rate across the Syrian population. The authors concluded that public awareness campaigns are required to increase the vaccine administration rate [12]. A recent systematic review mentioned a report about hesitancy to the COVID-19 vaccine in NWS, which showed high rejection rates to the vaccine (32% of the people completely rejected the idea of the vaccine, and 31% were hesitant) [22]. Hesitancy, the lack of resources, and insufficient planning were identified as the main challenges to improving the COVID-19 vaccine uptake in Syria [30]. Table 1 shows the potential reasons and their definitions for refusing a COVID-19 vaccination using standard terminologies.

**Table 1:** Potential reasons and their definitions for refusing the COVID-19 vaccination

Reasons for refusing a COVID-19 vaccine in NWS	Definition
Health concerns	Fear of side effects or possible vaccine-related disease or illness
Low subjective risk and carelessness	Underestimating the risk of the infection and the possibility of developing a severe course of the disease, especially among people who have already contracted the infection and recovered
Social norms	Estimating peers’ behavior and attitudes regarding the acceptance or refusal of the vaccine
Conspiracy theory	The belief that some covert but influential organizations or pharmaceutical companies are responsible for manufacturing the virus to increase their revenues from medicine and vaccine sales or that the vaccine contains genetically harmful materials
Spiritual and religious beliefs	The misconception of religious precepts that there is no infection and no evil omen and that the vaccination is against religious doctrines
Poor socioeconomic conditions	Limited access to financial, educational, social, and health resources affects people’s ability to interact and engage with the response plan (e.g., side effects might cause a person to be absent from work for several days and thus lose the source of daily income)
Disinformation and rumors	False information is deliberately and frequently spread covertly (as by rumors) in order to influence public opinion or obscure the truth, including misinformation from social media
Distrust or mistrust	Negative orientation or vigilance in whether the COVID-19 vaccine, service providers, Non-Governmental Organizations NGOs, and information are trustworthy
Ignorance of the existence of the disease or vaccine or the existence of vaccination campaigns in the area	Lack of knowledge or information about the disease and the available vaccination services

**Fig. 1:**
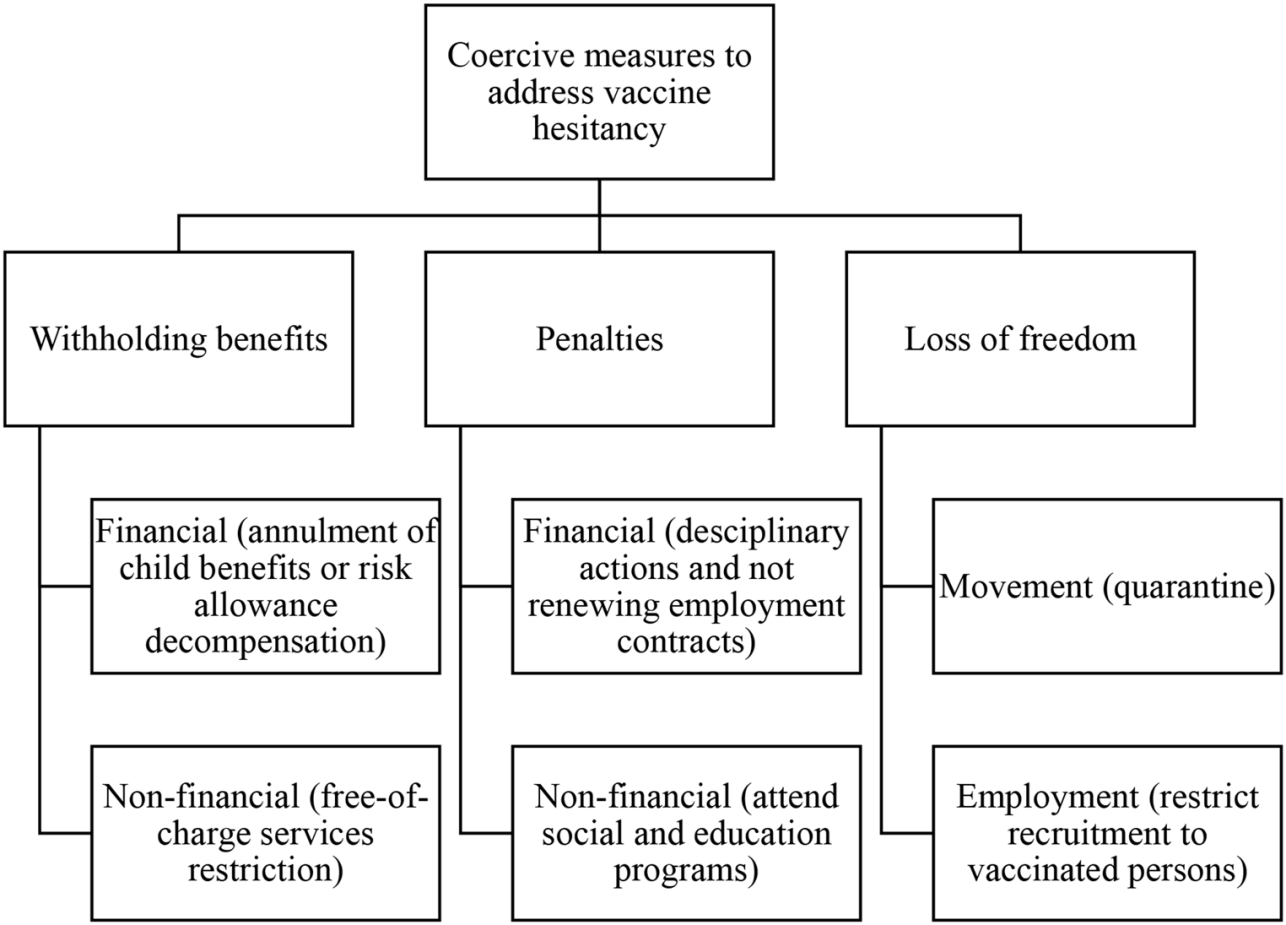
Coercive measures to address vaccine hesitancy through mandatory vaccination. Adapted from [40]

Strategies to address vaccine hesitancy are usually multi-modal because it is well-established that interventions based solely on the ‘knowledge-deficit’ model, which aims at improving individual knowledge, are insufficient to change vaccination behavior or boost vaccination confidence [31]. Synergized strategies, including medical workers’ and community leaders’ engagement, are vital to addressing hesitancy and promoting immunization [32]. Actions to address vaccine hesitancy could be categorized into coercive and persuasive measures (Verger and Dubé 2020). Public health ethics and law can justify the infringement of individual liberty in the name of protecting public health if they are proportionate and crucial [33, 34]. In recent years, some countries, in response to outbreaks of vaccine-preventable diseases, have expanded coercive action, making vaccination mandatory. This strategy, in fact, effectively increased vaccine coverage in many European countries [35, 36]. Nudging measures to promote vaccination might be administrative by restricting the recruitment of unvaccinated individuals or socioeconomic by not offering free-of-charge medical services to non-vaccinated individuals as a risk allowance or requesting documentation of immunization on school entry [35, 37]. Implementing coercive measures by humanitarian organizations is always problematic due to their commitment to humanitarian principles and compliance with the beneficiary protection approach [38]. According to Macklin (1989), coercive measures are that they force people to take risks against their personal judgment [39]. Savulescu (2021) categorized the coercive measures that constitute mandatory vaccination into; withholding benefits, penalties, and loss of freedom [40] (Fig. 1).

**Fig. 2:**
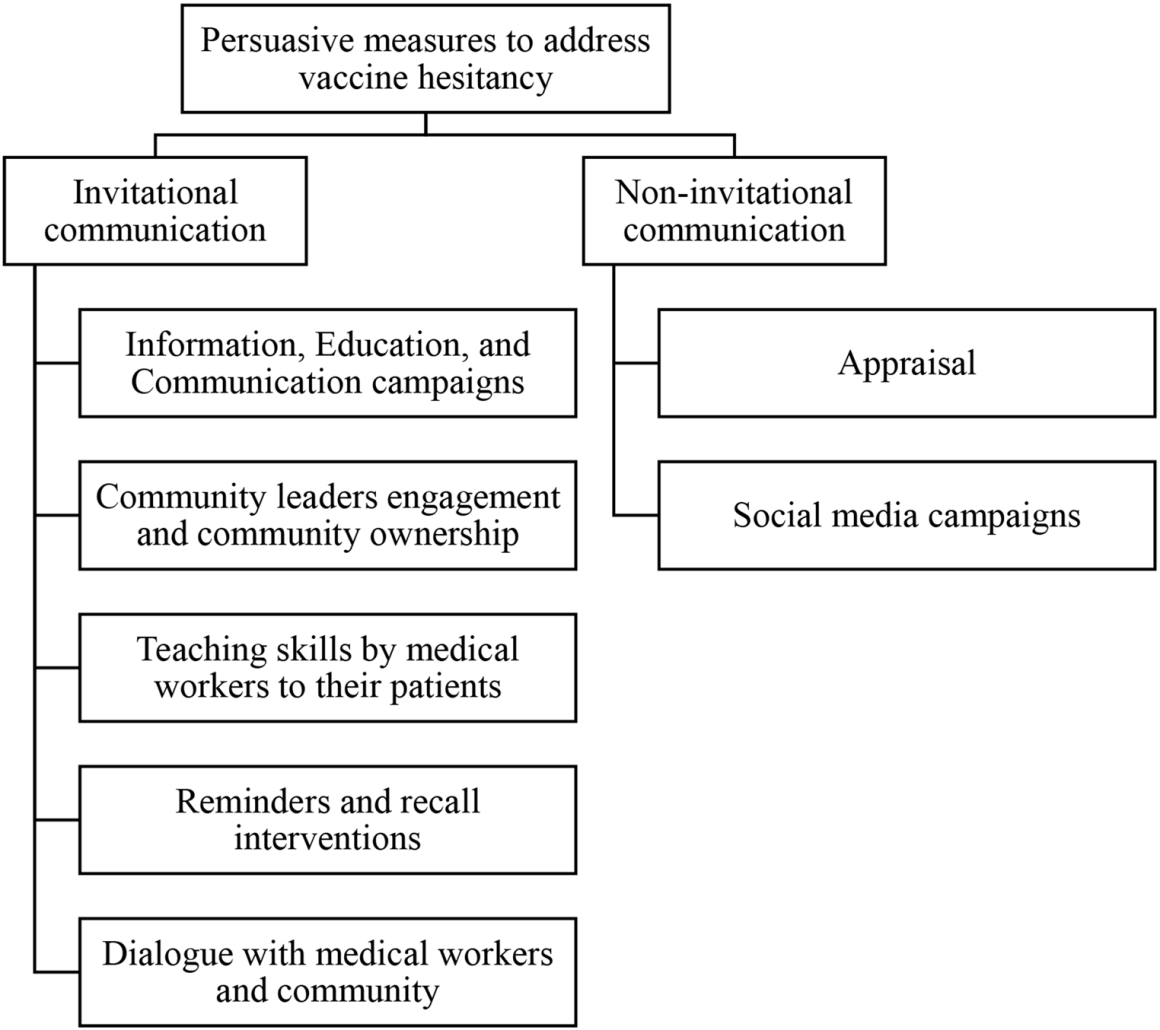
Persuasive measures to address vaccine hesitancy through invitational and non-invitational communication, adopted by the researchers from [50]

Persuasive measures, on the other hand, aim to change individuals’ attitudes, thoughts, and behaviors in favor of the vaccine [41]. Historically, traditional persuasive strategies for vaccine hesitancy included using fear-based messaging about the risks of not vaccinating and attempting to educate people about vaccinations by debunking vaccination myths and disseminating evidence-based information [42–45]. Many studies, however, established evidence that these traditional strategies, in some contexts, have a negative impact and might increase vaccine hesitancy because of the differing levels of scientific rhetoric and receiver’s knowledge and understanding, as well as the carelessness of people in protracted conflict and emergency contexts [46–48, 15]. Kempe et al. concluded that engaging health workers to boost vaccine uptake is a matter of time before they throw out of the task because at least 53% of medical workers spend an average of 15 min discussing the vaccine benefits with patients [49]. Lauver and Make (2022) categorized the persuasive measures to address vaccine hesitancy into; invitational (without inviting people to participate actively in the decision to accept the vaccine) and non-invitational (the use of individual or group motivational communication to rebut ambivalent messages and increase vaccine uptake) [50]. For this study, we adopted this definition to categorize persuasive measures to improve the COVID-19 vaccine acceptance rate in NWS (Fig. 2). These measures include RCCE or raising awareness campaigns based on solid evidence of the vaccine’s effectiveness and safety, appraisal and incentives, media campaigns, community leaders’ engagement and community ownership, teaching skills by medical workers to their patients, reminders and recall interventions, and dialogues [51–53].

Addressing vaccine hesitancy is challenging in conflict settings, especially with the absence of an administrative authority to enforce coercive measures, like in NWS [54]. COVID-19 vaccination figures in NWS are concerning and community engagement campaigns were ineffective in addressing hesitancy toward COVID-19 vaccines despite the remarkable resources invested in this regard [22]. This research aims to understand health behaviors and explore the challenges to improving the COVID-19 vaccine acceptance rates among the Syrian population in NWS, a context deeply affected by protracted conflict and political fragmentation, and investigate the measures WHO and SIG in Gaziantep– Türkiye, took to overcome these challenges, and conclude practical strategies to boost COVID-19 vaccine uptake in NWS. While existing research provides insights into vaccine hesitancy globally, there remains a significant gap in literature specifically addressing regions impacted by protracted conflicts, where conventional public health strategies may be less effective. The distinct sociopolitical context of NWS, characterized by its fragmented governance, collapsed health infrastructure, and diverse sociocultural dynamics, presents unique challenges that are not sufficiently captured in broader studies. This research aims to fill this critical gap by offering localized insights and evidence-based recommendations tailored to the NWS context. This study is the first to statistically study the relationship and correlation between the COVID-19 vaccine determinants and the outcome of concerns; accepting or rejecting the vaccine. The findings of this paper provide scientific evidence for decision-makers to amend their current strategies to contextually and practically address the low COVID-19 vaccine uptake in emergency and conflict settings, particularly in Syria.

## Methods and materials

The study employed a quantitative approach to examine COVID-19 vaccine hesitancy and uptake aiming to answer several questions related to the research topic in the context of NWS; (i) what are the reasons for COVID-19 vaccine hesitancy? (ii) What strategies were developed and implemented by humanitarian actors to address these reasons? (iii) Were these strategies effective in increasing vaccine acceptance and uptake? and (iv) What are the practical strategies for boosting vaccine uptake?

**Fig. 3:**
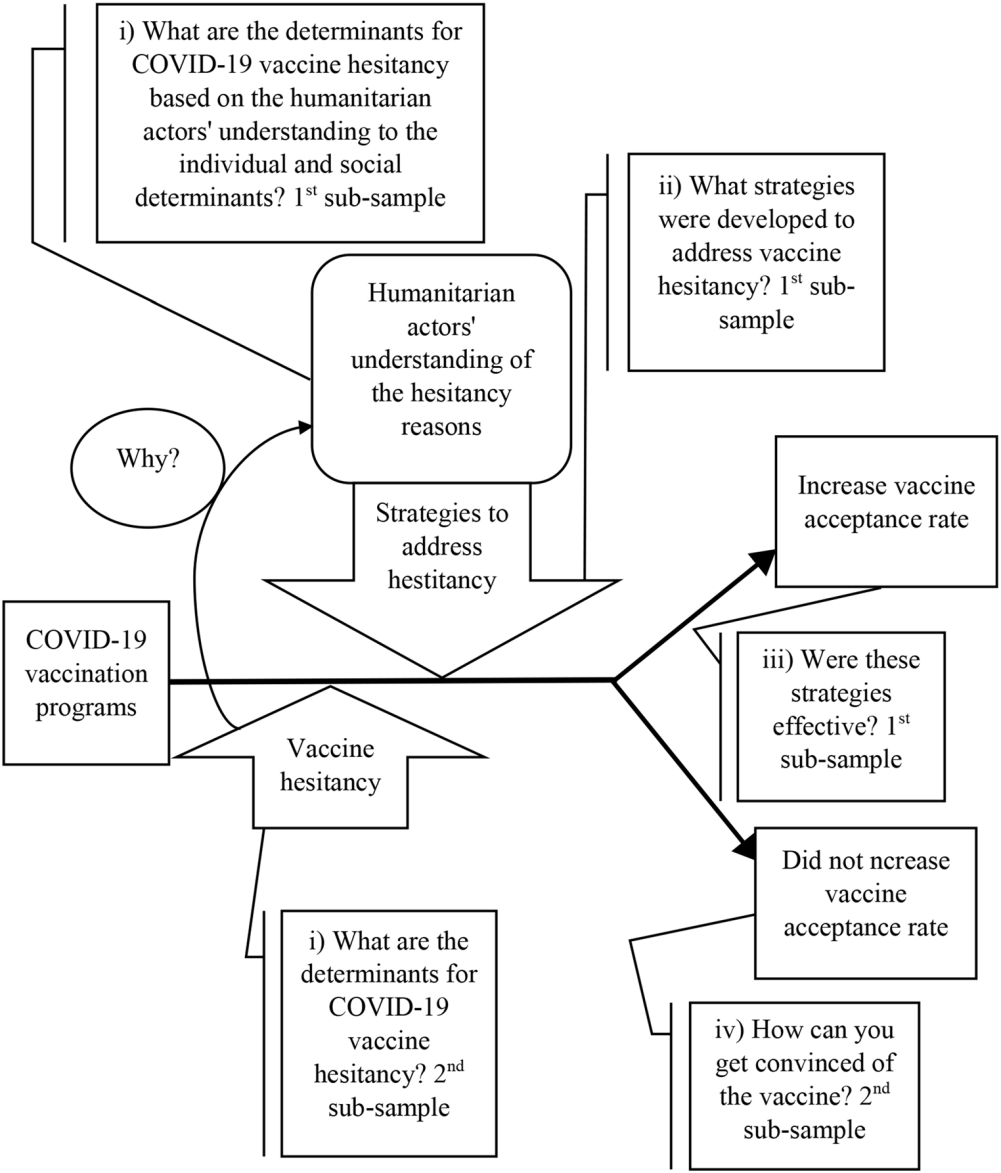
Research methodology and sampling division based on the research questions

Therefore, the sample was divided into two sub-samples (Fig. 3). The first was of the key decision makers and humanitarian workers from NGOs, UN agencies, local authorities, and SIG, based in Türkiye, and engaged in the COVID-19 outbreak response in NWS to investigate their knowledge of the hesitancy determinants and explore the plans set by these organizations to address these determinants. The second was of the people in NWS, i.e., those targeted with the vaccine, to identify the hesitancy reasons and determine practical policies to increase vaccine uptake. This design allowed for a comprehensive understanding from both the implementers’ and the recipients’ perspectives. Relevantly, two questionnaires and interview guides were developed in consultation with NGO workers and researchers to ensure the questionnaires include all potential challenges and strategies to the COVID-19 vaccination campaigns in NWS. The two questionnaires are available on the supplementary documents.

The first set of COVID-19 vaccine hesitancy determinant variables was selected based on the researchers’ knowledge and literature review and was subsequently expanded upon by feedback from SIG and WHO. The final set of variables to identify the COVID-19 vaccine hesitancy in NWS included sex, age, race, governorate, residential settings, education level, employment, and being a medical worker or not.

While an online Kobotool box was created for the first questionnaire and circulated by email and social communication tools, an in-person interview approach for the second survey was elected to encourage a free expression of views. A random sampling method was applied for the two sub-samples. Data were collected, in November and December 2022, by trained and qualified data collectors recruited from people in NWS to ensure community acceptance and interaction. Because medical workers are key in promoting or demoting vaccine uptake, doctors were hired and trained for data collection from medical workers to ensure smooth and scientific communication and interaction by this group. Besides, female data collectors were recruited to improve women’s responses and engagement. Consent was mandatory to participate in the research, and all data were anonymized and uploaded onto SPSS statistics 22 data analysis software program. The research framework, questionnaires, ethical clearance, consent form, NWS map, variable definition and values, anonymized row data, and data analysis extractions were uploaded to the Mendeley data website.

The validity of our survey was bolstered by a multidisciplinary team of experts in epidemiology, biostatistics, survey methodology, health communication, public health in emergencies, research ethics, and local language and cultural insights, ensuring that every aspect of the survey was tailored to the unique context of NWS.

### Statistical analysis

Descriptive analysis and frequency assessments were applied to the sociodemographic characteristics of the participants. The chi-square *χ*^*2*^ test was used to assess the association between nominal variables. Logistic regression was used to ascertain the correlation between the explanatory variables and the outcome of interest (accepting or rejecting the COVID-19 vaccine). In our logistic regression analysis, the dependent variable was the acceptance or rejection of the COVID-19 vaccine. The independent variables included a range of factors hypothesized to influence vaccine acceptance, such as age, gender, educational level, employment status, race, health concerns, misinformation, and socioeconomic conditions. Data analysis was performed using SPSS version 22. The significance level for all comparisons was set at a *p*-value of less than 0.05.

## Results

### Participants characteristics

The first survey targeted humanitarian workers and decision-makers from the humanitarian milieu in Türkiye– Gaziantep, where the humanitarian coordination mechanism for NWS cross-border emergency response is based [55]. Health workers comprising 5 females and 25 males from 30 NGOs, UN agencies, and other entities engaged in the COVID-19 outbreak preparedness and response plan participated in the first survey out of more than 50 active members and UN agencies within the health cluster [56]. Table 2 shows participants’ characteristics according to sex and type of organization. The majority of the participants, including the five females, were from NGOs: 21 participants.

**Table 2:** The first sub-sample participants’ characteristics (NGOs, UN agencies, SIG, and local authorities)

		Type of organization					Total
		Local authorities	NGOs	Other	SIG	UN agency	
Sex of participants	female	0	5	0	0	0	5
	male	2	16	1	3	3	25
Total		2	21	1	3	3	30

A total of 434 individuals participated in the second survey (37. 6% females and 62.4% males). The mean age of the participants was 35.5 years. Two ethnicities participated in the survey, Arabs (84%) and Kurds (16%), and the majority of the participants (61%) were from Aleppo governorate. Almost 36.2% of the participants were IDPs living in camps or informal residency settings. While 12.2% of the participants were illiterate, about 51% had primary, preparatory, or secondary education certificates, and 36.8% had intermediate or university education certificates or higher. Besides, almost 15% of the participants were medical workers (doctors, dentists, pharmacists, medical technicians, midwives, and nurses). Disaggregated information about the 2nd survey participants’ characteristics is shown in Table 3.

**Table 3:** The second sub-sample participants’ characteristics

Sex of participants	Race of participants		Governorate		Residential setting		Education level				Are you a medical worker?		Total
	Arabic	Kurdish	Aleppo	Idleb	Camp or informal setting	Formal residential setting	Illiterate	Secondary school education and lower	University and higher education or Intermediate Institute	No		Yes	
Male	214	57	173	98	168	103	38	140	93	55		40	271
Female	150	13	92	71	109	54	15	81	67	50		25	163
Total	364	70	265	169	277	157	53	221	160	105		65	434

The majority of the participants confirmed that they had encountered information about the disease (98.6%) and vaccines (97%). However, more than 36% of the participants do not believe the disease is risky or life-threatening, and 20% said they do not know there are COVID-19 vaccine campaigns in their areas.

### Reasons for the COVID-19 vaccine hesitancy (question i)

Almost all the participants of the first survey confirmed the high rates of COVID-19 vaccine hesitancy in NWS. When the participants of the first survey were asked about the reasons for COVID-19 vaccine hesitancy (a multiple-choice question), the reasons were mostly related to fear of side effects, social media misinformation, carelessness, and conspiracy theories, like the vaccine is against the culture and gender (Table 4). Social norms and religious beliefs were not among the most frequent reasons based on the findings of the first survey. Almost 10% of the respondents linked vaccine refusal with the lack of education and health awareness among people in NWS.

**Table 4:** Reasons for COVID-19 vaccine hesitancy in NWS according to the participants of the first sub-sample (NGOs, UN agencies, SIG, and local authorities)

		Responses		Percent of Cases
		N	Percent	
Reasons	Health concerns (e.g., fear of side effects)	26	20.3%	86.7%
	Disinformation and rumors (social media)	23	18.0%	76.7%
	Low subjective risk and carelessness	21	16.4%	70.0%
	Conspiracy theory	16	12.5%	53.3%
	The lack of education and health awareness	13	10.2%	43.3%
	Poor socioeconomic conditions	11	8.6%	36.7%
	Social norms	7	5.5%	23.3%
	Insufficient planning and resources	9	7.0%	30.0%
	Spiritual and religious beliefs	2	1.6%	6.7%
Total		128	100.0%	426.7%

Out of the total participants of the second sub-sample, 241 (55.5%) refused the vaccine (zero doses), 63 (14.5%) were partially vaccinated, and 130 (30%) were fully vaccinated. The hesitancy reasons not to take the COVID-19 vaccine in NWS based on the second survey participants varied noticeably (Table 5) but were mainly related to Low subjective risk and carelessness (28.8%), health concerns (24.4%), disinformation and rumors (15.4%), and distrust or mistrust (13%). The main reasons for the refusal of the vaccine by medical workers were low subjective risk and carelessness, followed by distrust of the vaccine. However, there was no significant relationship between being a medical worker and refusing the vaccine (*p* = 0.467, CI: 95%).

**Table 5:** Reasons for COVID-19 vaccine hesitancy in NWS according to the participants of the second sub-sample

		Responses		Percent of Cases
		N	Percent	
Hesitancy reasons	Low subjective risk and carelessness	86	28.8%	36.0%
	Health concerns and fear of side effects	73	24.4%	30.5%
	Disinformation and rumors	46	15.4%	19.2%
	Distrust or mistrust	39	13.0%	16.3%
	Conspiracy theory	29	9.7%	12.1%
	Ignorance or lack of information about the disease, the vaccine, or the existence of vaccination campaigns	16	5.4%	6.7%
	Poor socioeconomic conditions	7	2.3%	2.9%
	Spiritual and religious beliefs	2	0.7%	0.8%
	Social norms	1	0.3%	0.4%
Total		299	100.0%	125.1%

Although the first six reasons were significantly related to refusing the COVID-19 vaccine (*p* < 0.05, CI: 95%), we could not find a linear model of correlation between these reasons and rejecting the vaccine.

When the percentages of people who refused the vaccine were compared according to their educational level (Table 6), it was found that most illiterate and those with secondary education or less (67.9%, and 67.4%, respectively) refused to take the vaccine. When studying the correlation between the two variables using logistic regression, it was found that education level was significantly related to refusing the vaccine when using people with university or higher education as a reference group (χ2 = 43.26, *p* < 0.05, CI: 95%). The odds ratio exp(B) value indicates that illiterate people and those with a secondary education level and lower had almost 0.25 odds of accepting the vaccine than the reference group (Table 7).

**Table 6:** Crosstabulation of refusing the COVID-19 vaccine and education level among the second sub-sample

			Have you received the COVID-19 vaccine?		Total
			No	Yes	
Education level	Illiterate	N	36	17	53
		%	67.9%	32.1%	100%
	Secondary school education and lower	N	149	72	221
		%	67.4%	32.6%	100%
	University and higher education or Intermediate Institute	N	56	104	160
		%	35%	65%	100%
Total		N	241	193	434
		%	55.5%	44.5%	100%

**Table 7:** Correlation between refusing the COVID-19 vaccine and education level among the second sub-sample (the outcome of preference is: accepted to receive the COVID-19 vaccine)

Education level	B	S.E.	Wald	df	Sig.	Exp(B)	95% C.I.for EXP(B)	
							Lower	Upper
University and higher education or Intermediate Institute			41.364	2	0.000			
Secondary school education and lower	-1.369	0.338	16.438	1	0.000	0.254	0.131	0.493
Illiterate	-1.346	0.219	37.705	1	0.000	0.260	0.169	0.400

Logistic regression showed a significant correlation between race and vaccine refusal. Kurdish people are less likely to accept the vaccine than Arabs, with an exp(B) of 0.374 (*p* < 0.05, CI: 95%) to receive the COVID-19 vaccine. The percentage of Kurdish people who received the vaccine (25.7%) was less than that of Arabic (48.1%). In addition, employment was significantly related to refusing the COVID-19 vaccine in NWS. Unemployed people were less likely to accept the vaccine than employed people, with exp(B) = 0.296 (*p* < 0.05, CI: 95%).

Variables like sex, age, governorate, and residential settings were not significantly related to the outcome of interest, refusing or accepting the COVID-19 vaccine in NWS.

### Strategies to increase the COVID-19 vaccine uptake (questions ii & iii)

Out of the 30 participants in the first survey, 26 mentioned that their organizations were directly or indirectly involved in the COVID-19 vaccination campaigns by implementing programs that aim to increase COVID-19 vaccine uptake in NWS, such as resources mobilization, vaccination service delivery, community engagement, and raising awareness. The participants were asked about strategies implemented by their organizations to address COVID-19 vaccine hesitancy and increase community trust. These strategies were categorized into coercive and persuasive measures based on the aforementioned classification (Figs. 1 and 2). In fact, the majority of the measures implemented by humanitarian organizations were persuasive, such as RCCE and social media campaigns, community leaders’ engagement and community ownership, and teaching skills by medical workers to their patients. Two participants mentioned that their organizations applied financial and behavior-based appraisal strategies to enhance vaccine uptake (Table 8). Besides, invitational persuasive strategies to address COVID-19 vaccine hesitancy among NWS populations were the most forth (78% of the responses about strategies to address COVID-19 vaccine hesitancy).

**Table 8:** Coercive and persuasive strategies to address COVID-19 vaccine hesitancy according to the participants of the first survey (NGOs, UN agencies, SIG, and local authorities)

Strategies to address the COVID-19 vaccine hesitancy		Responses		Percent of Cases
		N	Percent	
	RCCE campaigns	20	33.9%	74.1%
	Appraisal and incentives	2	3.4%	7.4%
	Dialogue with medical workers	5	8.5%	18.5%
	Community leader’s engagement and community ownership	8	13.6%	29.6%
	Social media campaigns	10	16.9%	37.0%
	Teaching skills by medical workers to their patients	8	13.6%	29.6%
	Dialogue and group discussions with the community	5	8.5%	18.5%
	Withholding benefits	1	1.7%	3.7%
Total		59	100.0%	218.5%

Information from the second survey clearly pointed out the high hesitancy rate among people in NWS. Most of the strategies mentioned by the participants of the first sub-sample were not recognized by the participants of the second sub-sample based on their answers. Only 47 (10.8%) participants in the second survey were against the vaccine but took it later. Of them, 42 answered the question of how or why they changed their convection to take the vaccine. The majority of the respondents to this question said that they had to take the vaccine due to coercive measures (45%) by employers (like NGOs). Only 26.2% said they changed their convictions about the vaccine due to persuasive measures like RCCE campaigns, even though these campaigns were among the top three sources of information about the vaccine in NWS. In fact, 15.8% of the participants said they had received information that might be convincing about the vaccine from community workers through RCCE campaigns. What was remarkable about the results of the study was that none of the participants in the second sub-sample who changed their opinion to take the vaccine recognized teaching and awareness messages by medical workers as a reason (Table 9).

**Table 9:** Reasons for accepting the COVID-19 vaccine after refusing it among the second sub-sample

How or why did you change your conviction about the vaccine?		Frequency	Percent	Valid Percent
	Fear of disease consequences	7	1.6	16.7
	Forced for job or travel	19	4.4	45.2
	From the positive experience of people who received the vaccine	5	1.2	11.9
	RCCE	11	2.5	26.2
	Total	42	9.7	100.0
Missing	System	392	90.3	
Total		434	100.0	

While 45.9% of the participants said that the information they encountered was more convincing about the importance of the vaccine, 13.1% of them said that the information that was against the vaccine was more credible, and 27.9% said that they came across influential information for both purposes (13.1% of the participants did not answer this question). The leading sources of information were Facebook and social media (28.8%), medical workers (20.6%), and social and community workers (15.8%). The participants mentioned other sources of information like road signs of the SIG social mobilization campaigns, family members and relatives, and key community people like teachers and Islamic clerics. However, it was found that among these sources, only receiving information from medical workers was significantly related to taking the vaccine as an outcome (χ2 = 37.945, *p* < 0.05, CI: 95%) (Table 10). Based on these findings, people are 3.2 times more likely to accept the vaccine if they were subjected to risk communication and awareness about the vaccine from medical workers compared with other sources of information.

**Table 10:** Correlation between accepting3 the COVID-19 vaccine and source of information about the vaccine in the second sub-sample (the outcome of preference is: accepted to receive the COVID-19 vaccine)

Source of information about the COVID-19 vaccine	B	S.E.	Wald	df	Sig.	Exp(B)	95% C.I. for EXP(B)	
							Lower	Upper
Medical workers	1.175	0.226	27.152	1	0.000	3.239	2.082	5.039
Facebook and social media	0.233	0.264	0.779	1	0.378	1.263	0.752	2.119
Social and community workers (RCCE programs)	0.068	0.241	0.080	1	0.778	1.070	0.667	1.718
Road signs of the social mobilization campaigns (RCCE programs)	− 0.116	0.244	0.223	1	0.636	0.891	0.552	1.439
One of the family members or relatives	0.052	0.222	0.056	1	0.813	1.054	0.682	1.629
Key people in the community, like Islamic clerics or teachers (RCCE programs)	− 0.353	0.275	1.647	1	0.199	0.702	0.410	1.205

### Methods to promote people’s perspectives on the COVID-19 vaccine in NWS (question iv)

The participants who refused the vaccine (55.5%) were asked about the ways in which they could be persuaded to engage in the RCCE campaigns and accept the vaccine. Indeed, 24% of the respondents said they would not change their perspectives on the vaccine, no matter what method. The binary logistic regression analysis showed a significant correlation between reluctance to discuss methods of persuasion with the vaccine and governorate (χ2 = 11.272, *p* < 0.05, CI: 95%). Residents from Aleppo are more likely to reject discussing ways of convincing them about the vaccine. Almost 22.5% of the respondents indicated their need for more information and scientific evidence about the disease to think about accepting the vaccine because they doubt the rapid emergence and spread of the virus and the short period it took to produce the vaccine, which contradicts what they know about other vaccines. The use of vaccines without or with mild side effects (12.7%), receiving advice from medical workers (11.8%), and witnessing the positive experience of people who received the vaccine (11.8%) were among the methods that might convince the people to receive the vaccine. The methods suggested by the second sub-sample to effectively engage them varied between the medical and non-medical workers. Almost 44% of the medical workers considered providing scientific information about the disease and vaccine through dialogue sessions an effective strategy. This strategy was the second one in order (17.5%) after using vaccines without or with mild side effects (20%), followed by receiving advice about the vaccine from a medical worker (12.5%) based on the response of the other respondents in the second survey.

## Discussion

The research aims at finding answers to several questions to investigate the low uptake of the COVID-19 vaccine in NWS and conclude contextual and practical solutions. According to the second survey results, 55.5% of the respondents refused the COVID-19 vaccine. The research findings indicate a similarity between the identified reasons by the first sub-sample regarding the low vaccination rate (Table 4) and the real reasons according to the answers of the second sub-sample (Table 5), more precisely, the top three reasons (low subjective risk and carelessness, health concerns including fear from side effects, and disinformation and rumors). Race, employment, and low level of education were significantly related to vaccine refusal. People with less than a university education, Kurds, or non-employed persons were more likely to refuse the vaccine. The place of residency, displacement, or medical nature of the job did not determine vaccine refusal. Sex was not related to vaccine hesitance, even when confounded by other variables like education or residency settings (camps or formal residency settings). Although several articles [57–59] have addressed the high COVID-19 vaccine hesitancy among Kurdish people in neighborhood countries, comparing the results of these studies to the findings of this research might be inapplicable due to variance in terms of existing socioeconomic and geopolitical factors.

The research findings indicate a knowledge gap and lack of evidence regarding strategies of humanitarian actors to address the low uptake of the COVID-19 vaccine and the impact of these strategies. When respondents in the first sub-sample (humanitarian actors) were asked whether these strategies were effective in addressing COVID-19 vaccine frequency, 70% said these strategies were effective, which appears to contradict their assertion of the high rate of vaccine hesitancy in NWS despite the apparent congruence between the reasons identified by the first sub-sample regarding the refusal of the vaccine and the real reasons. By diving deep into the findings of this question, it was found that all those who said there is vaccine hesitancy but confirmed that the strategies to address this hesitancy were effective despite the low acceptance rate were from NGOs, SIG, and local authorities. Moreover, the research results showed that strategies implemented by humanitarian actors (Table 8) to address vaccine hesitancy, including RCCE as one of the most implemented activities, were ineffective, as they contributed to persuading only 10.8% of the participants in the second sub-sample to accept the vaccine. In addition, information from RCCE programs was neither associated nor correlated with increasing vaccine uptake (Table 10). Many articles argued that RCCE, as one of the leading strategies in response to the COVID-19 outbreak and vaccine promotion plan, was ineffective in NWS because it was not comprehensive, not evidence-based, and not contextualized to the protracted crisis and the geopolitical and economic situation in Syria [15, 60]. Participants in the second sub-sample did not recognize teaching skills by doctors and medical workers as a persuasive approach to vaccine acceptance. However, it was statistically revealed that doctors were a reliable source of information for people to accept the vaccine. Although this strategy, teaching skills by medical workers to their patients, was not among the most implemented by humanitarian actors (Table 8), it could contribute significantly to increasing the vaccine uptake in NWS.

According to the participants who refused the COVID-19 vaccine, methods of convincing them to accept the vaccine were persuasive, mainly by communicating scientific information and evidence about the virus and the safety of the vaccine. Doctors and medical workers considered this approach as the most effective in approaching them. The results of this research align with findings from various studies conducted in low and middle-income countries. These studies have demonstrated that persuasive strategies are highly effective in encouraging public participation in health campaigns, particularly when these strategies are underpinned by a comprehensive analysis of factors contributing to hesitancy and are based on a contextual understanding of the prevailing circumstances [61–63]. Besides, health concerns and fear of side effects were among the most prominent reasons to reject the COVID-19 vaccine despite the available access to health services. When studying the other methods mentioned in the second sub-sample, it was clear that there are complementary links between them. Nearly a quarter of the sampled people who refused the vaccine indicated that they would accept it if a vaccine brand with minor or no side effects was used and if they witnessed safe experiences for people who received the vaccine. These two methods are largely interrelated, as the use of vaccines with almost no side effects results in a safe vaccination experience, which might contribute to disseminating positive information [64, 65]. It is estimated that experiencing and witnessing severe or moderate side effects prevented 24.4% of people in NWS from accepting the vaccine. This fact highlights the importance of programming vaccination campaigns in terms of quality assurance and selecting target people in a way to minimize developing side effects and consequently avoid promoting the spread of disinformation and rumors. Additionally, approaching people by medical workers with awareness messages about the vaccine was found to be one of the preferred strategies by more than 11% of the people in NWS. The topic of programming vaccination campaigns indeed needs to be further investigated and researched in protracted emergency settings. With this respect, follow-up research has been initiated by the research group to examine the effects of dialogue sessions, a resolution derived from this study, aimed at assessing the attitudes of medical professionals participating in these sessions in NWS towards COVID-19 vaccines and how this will influence people’s attitudes to COVID-19 vaccination.

The study showed that 24% of the participants would not change their opinion of refusing the vaccine, regardless of the strategy used to convince them. The governorate of residence was significantly related to the participants’ absolute refusal to be convinced of the vaccine. This bizarre relationship opens the door to new questions about what is different in the vaccination strategies between the governorates and if hidden variables influence people’s perspectives on the vaccine. Besides the different political powers of control, the noticeable difference between the two governorates is the discrepancy in the governance system of the health sector. Idlib Health Directorate is the central body for governance and monitoring of the health system in Idleb, unlike the case in Aleppo governorate, which lacks centralized health governance and the presence of systematic field coordination due to the multiple district health directorates in the governorate [66–68].

It is worth noting that the constrained engagement of humanitarian entities, including International NGOs and UN agencies, posed a significant challenge in capturing a holistic picture of COVID-19 vaccine hesitancy in NWS. Additionally, the limited participation of women in crucial research issues related to NWS is still an obstacle that limits the holistic view and solutions to these issues. Although the first questionnaire reached large segments of decision-making positions of both sexes, women’s participation constituted less than a quarter of all participants. Therefore, saying that the results of this research are somewhat limited at the level of decision-making from a holistic view of both sexes might be a valid argument. Based on the lessons learned from this research, we recommend that researchers who intend to conduct scientific studies in Syria, and other conflict areas similar in demographic structure and culture, plan practically to reach results representative of both sexes. Additionally, The self-reported nature of vaccine uptake introduces the potential for bias, particularly towards false positives, as this behavior aligns with the expectations the researchers may have of the interviewees.

## Conclusion

The research aims to investigate the correlation between public health determinants and accepting or rejecting the COVID-19 vaccine. Humanitarian actors must reconsider their strategies to address vaccine hesitancy in NWS. The research results indicate the importance of targeting medical workers with strategies distinct from those used with others. These strategies must be based on a persuasive approach with regard to convincing them not of the benefits or necessity of the vaccine but rather of the fact of the existence of the epidemic and the mechanism of developing the vaccine in an evidence-based and scientific way in order to ensure that they are recruited intellectually and practically with plans for vaccine campaigns as community mobilizers.

The current RCCE program is not a key to increasing vaccine uptake in NWS. Information disseminated by the community and social workers through the RCCE program must be reassessed to address the hesitancy determinants besides the vaccination benefits. Vaccination campaigns must be reprogrammed through evidence and information about the reasons for refusing the vaccine and methods of effective risk communication and targeting influential groups of society such as doctors and health workers and engaging them systematically in vaccination plans and community mobilization. The role of health workers in influencing people’s perspectives on the vaccine must be further researched to develop a context-specific and applicable engagement plan for medical workers.

A comprehensive study of the determinants of vaccine refusal in the NWS should be undertaken to identify barriers to the effective inclusion of minorities and vulnerable people on the basis of race and education and to uncover confounding variables that may influence people’s views of the COVID-19 vaccine.

### Electronic supplementary material

Below is the link to the electronic supplementary material.

Supplementary Material 1

Supplementary Material 2

## Data Availability

The datasets generated and/or analyzed during the current study are available on the Mendeley Data website https://data.mendeley.com/datasets/8g9g633j9f.

## Abbreviations

HTS: Hay’at Tahrir al-Sham
NGOs: Non-Governmental Organizations
NWS: Northwest Syria
RCCE: Risk Communication and Community Engagement
SIG: Syria Immunization Group
UN: United Nations
UNICEF: United Nations Children’s Fund
UNSCR: United Nations Security Council Resolution
WHO: World Health Organization
