# Supplementary material for: Individual and social determinants of COVID-19 vaccine hesitancy and uptake in Northwest Syria
Source: BMC Health Serv Res. 2024 Mar 1;24:265. doi: 10.1186/s12913-024-10756-z (PMC10908183; doi:10.1186/s12913-024-10756-z)
Supplement: Supplementary file 1 — Supplementary Material 1 [file 12913_2024_10756_MOESM1_ESM.pdf]

Strategic Research Center
SRC

Questionnaire #1 to UN, NGOs, SIG, and local authorities.

Research Title: **Individual and Social Determinants of COVID-19 Vaccine Hesitancy and Uptake in Northwest Syria**

Ethical approval number: 05/2022

Dear Participant,

Thank you for taking the time to read and answer this questionnaire. This questionnaire is part of a research study that aims to identify the reasons behind the COVID-19 vaccine hesitancy among the populations of NWS and how the current immunization campaign has addressed community concerns regarding the COVID-19 vaccine.

The research is undertaken by the Strategic Research Center (Öz SRC), Gaziantep – Turkey, registration number: 53860.

Your participation in this study is voluntary. It is up to you to decide whether or not to take part in this study. If you decide to participate in this study, you will be navigated to the questionnaire after accepting the consent statement. You are still free to withdraw at any time and without giving a reason. Withdrawing from this study will not affect the relationship you have, if any, with the researcher. If you withdraw from the study before data collection is completed, your data will be abolished.

The answers will be stored and secured on a safe and inaccessible server. Data could be used later for research purposes only.

If you have any questions before participating in the questionnaire, please do not hesitate to email us at info@strategicresearchcenter.com

The expected time to complete the questionnaire is 5 minutes.

To participate in the questionnaire, please read the following statement and click on YES to start to answer the questions.

I have read and understand the provided information and have had the opportunity to ask questions. I understand that my participation is voluntary and that I am free to withdraw at any time, without giving a reason and without cost. I understand that I will be given a copy of this consent form. I voluntarily agree to take part in this study. **YES – No**

Do not proceed with the questions if the answer is (No).

1- Sex (male – female)
2- Place of work (UN agency, NGO, Syrian Immunization Group SIG, local authority)
3- Was your organization/institute/agency involved in the COVID-19 outbreak preparedness and response? (yes – no)
4- Do you agree that there is a high hesitancy to the COVID-19 vaccines in NWS? (yes – no)
5- What are the main reasons for the COVID-19 vaccine hesitancy in NWS?

6- Was your organization/institute/agency involved in the COVID-19 vaccination campaigns (administration, implementation, planning, raising awareness, risk communication and community engagement, logistics, or financial support)? (yes – no)
7- What strategies were developed by your agency or in partnership with other agencies or NGOs to address these reasons?
8- Do you think these strategies were effective in increasing the COVID-19 vaccine uptake in Northwest Syria? (yes – no)
