# Supplementary material for: Individual and social determinants of COVID-19 vaccine hesitancy and uptake in Northwest Syria
Source: BMC Health Serv Res. 2024 Mar 1;24:265. doi: 10.1186/s12913-024-10756-z (PMC10908183; doi:10.1186/s12913-024-10756-z)
Supplement: Supplementary file 2 — Supplementary Material 2 [file 12913_2024_10756_MOESM2_ESM.pdf]

Strategic Research Center
SRC

Questionnaire 2 to people and medical workers in Northwest Syria

Individual and Social Determinants of COVID-19 Hesitancy and Uptake in Northwest Syria

1- ID:
2- Sex (male-female)
3- Age (years):
4- Race: (Arabic – Kurdish – Turkman – Other).
5- Place of current residence: (governorate – community). *(E.g., Aleppo – A'zaz)*
6- Current residential setting: (camp or informal setting – formal residential setting).
7- Level of education (university and higher education or Intermediate Institute, secondary school education and lower, illiterate).
8- Employed (yes – no)
    a. If yes, are you a medical worker?
        i. If yes, what is your current profession (doctor, dentist, pharmacist, nurse or paramedic, midwife, medical technician, medical assistant)?
        ii. If not, what is your current profession?
9- Have you ever heard about COVID-19 or coronavirus? (yes – no)
10- Do you think that it is a risky or life-threatening disease? (yes – no)
11- Have you ever heard about the COVID-19 vaccine? (yes – no)
12- Do you know that there is a COVID-19 vaccination campaign in the region? (yes – no)
13- Have you received the COVID-19 vaccine? (yes – no)
    a. If yes
        i. How many doses (1 – 2 or more)
            - If one does, why didn't you take the 2nd dose? (not yet, reject)
              If rejected, ask question 13.b.i, and do not ask it later.
        ii. Were you against the COVID-19 vaccine and changed your mind? (yes – no)
            - If yes, why?
    b. If not
        i. Why have you not received the COVID-19 vaccine?
        ii. How could you get convinced that the vaccine is necessary and safe?
14- Have you ever encountered information about the COVID-19 vaccine? (yes – no)
    a. If yes, what was the source *(you can choose more than one answer)*? (medical workers, Facebook and social media, social and community workers, road signs, family, and key people in the community like a sheikh or teacher)
    b. Was the information for or against the COVID-19 vaccine?

Strategic Research Center
SRC

المحددات الفردية والاجتماعية المرتبطة برفض وقبول لقاح الكوفيد-19 في منطقة شمال غرب سورية

1- الرقم
2- الجنس (ذكر – أنثى)
3- العمر (بالسنوات):
4- العرق (عربي – كردي – تركمان – أخرى)
5- مكان السكن الحالي (السكن – المنطقة) *(مثال: حلب – أعزاز)*
6- طبيعة السكن الحالي: (مخيمات أو مراكز اللجوء أو إيواء النازحين – سكن رسمي)
7- مستوى التعليم (جامعي أو معهد متوسط – شهادة ابتدائية أو إعدادية أو ثانوية – أمي)
8- موظف (لديك وظيفة حالياً): (نعم – لا)
   a. في حال كان الجواب نعم، هل أنت موظف في المجال الطبي؟ (نعم – لا)
      i. في حال كان الجواب نعم، ما هي مهنتك الحالية (طبيب بشري – طبيب أسنان – صيدلاني – ممرض أو مسعف – قابلة – فني طبي – مساعد تمريض أو مساعد طبي)
      ii. في حال كان الجواب لا، ما هي وظيفتك الحالية؟
9- هل سمعت مسبقاً بمرض الكورونا أو الكوفيد-19؟ (نعم – لا)
10- هل تعتقد أن هذا المرض خطير أو قد يهدد الحياة في بعض الحالات؟ (نعم – لا)
11- هل سمعت مسبقاً بلقاح الكورونا أو لقاح الكوفيد-19؟ (نعم – لا)
12- هل تعلم بأنه يوجد حملة لقاح كورونا أو لقاح كوفيد-19 في منطقتك؟ (نعم – لا)
13- هل تلقيت لقاح الكوفيد-19؟ (نعم – لا)
   a. في حال كان الجواب نعم:
      i. كم عدد الجرعات التي تلقيتها؟ (1 – 2 أو أكثر)
         - إذا كان الجواب جرعة واحدة، لماذا لم تأخذ جرعة ثانية؟ (لم يحن الموعد – رفض)

         في حال كان الجواب رفض، اسأل السؤال 13.b.i ولا تسأله لاحقاً.
      ii. هل كنت ضد لقاح الكوفيد-19 وغيرت رأيك حين أخذت اللقاح؟ (نعم – لا)
         - في حال كان الجواب نعم، لماذا أو كيف غيرت رأيك؟
   b. في حال كان الجواب لا:
      i. لماذا لم تقبل أن تأخذ لقاح الكوفيد-19؟
      ii. كيف يمكن أن تقتنع بأن اللقاح ضروري وآمن؟
14- هل تعرضت أو صادفت أو شاهدت مسبقاً معلومات عن لقاح الكوفيد-19؟ (نعم – لا)
   a. في حال كان الجواب نعم، ما هو مصدر المعلومات *(يمكن اختيار أكثر من إجابة)*؟ (أشخاص من المجال الطبي كأطباء وممرضين، الفيس بوك ووسائل التواصل الاجتماعي، عمال الصحة المجتمعية، لافتات طرقية، العائلة، أشخاص مهمين في المجتمع كإمام المسجد أو شيخ أو معلم)
   b. هل كانت المعلومات تنصح بلقاح الكوفيد-19 أو ضد لقاح الكوفيد-19
